# Spontaneous Pneumothorax, Pneumomediastinum, and Pneumopericardium in an HIV Patient With Tuberculosis: A Rare Trio

**DOI:** 10.7759/cureus.58440

**Published:** 2024-04-17

**Authors:** Jithesh G, Swetha Narayanan, Sahil Kumar, Madhav Banjade, Mukesh Bairwa

**Affiliations:** 1 Internal Medicine, All India Institute of Medical Sciences, Rishikesh, Rishikesh, IND; 2 Hospital Medicine and Critical Care, Internal Medicine, All India Institute of Medical Sciences, Rishikesh, Rishikesh, IND

**Keywords:** tamponade pneumopericardium, secondary pneumomediastinum, pneumothorax (ptx), opportunist infections in hiv, human immunodeficiency virus (hiv) infection

## Abstract

A trio of spontaneous pneumomediastinum, pneumopericardium, and pneumothorax is a highly unusual presentation. The majority of reported cases are due to trauma, while the remaining cases are iatrogenic. Among infections, this trio has so far been reported in COVID-19 pneumonia and pneumocystis pneumonia in HIV-positive patients. There are case reports on pneumothorax and pneumomediastinum in tuberculosis, but the trio is not reported. Here, we present a case of a recently diagnosed HIV-positive patient with complaints of cough and shortness of breath whose initial workup was negative for Mycobacterium. The patient was, however, started on antitubercular drugs based on clinical radiological evidence. He developed spontaneous pneumothorax, pneumomediastinum, and pneumopericardium, and repeat bronchoalveolar lavage (BAL) came positive for Mycobacterium. The patient, however, could not be revived and succumbed to obstructive and septic shock.

## Introduction

Spontaneous pneumothorax is a frequently reported complication in HIV patients, occurring due to various etiologies, particularly infections such as Pneumocystis carinii pneumonia (PCP), bacterial pneumonia, and pulmonary tuberculosis (TB), as well as toxoplasmosis, fungal, viral, and atypical mycobacterial infections [1-3]. The common organism reported to cause pneumonia and concurrent pneumomediastinum is P. carinii. Only one of the identified cases was caused by cytomegalovirus. However, the combination of pneumothorax and pneumomediastinum is reported only in P. carinii pneumonia. [3] In this case report, we present the trio of spontaneous pneumothorax, pneumomediastinum, and pneumopericardium in a recently diagnosed HIV patient with a very low CD4 count who was also diagnosed with pulmonary TB.

## Case presentation

A 36-year-old male presented with a history of intermittent low-grade fever for a one-year duration associated with unintentional weight loss of 15 kg in six months, cough with minimal expectoration for one month associated with progressive shortness of breath, and pleuritic chest pain. There were no prior comorbidities, addictions, or contact with TB patients. For these complaints, the patient was admitted to another hospital and was diagnosed with HIV seropositive. He was started on antitubercular drugs (isoniazid, rifampicin, pyrazinamide, and ethambutol) based on clinicoradiological evidence and discharged on antitubercular treatment (ATT). Sputum workup was negative for acid-fast bacilli. Two days before presentation to our hospital, the patient developed an altered sensorium. On general examination, the patient had pallor and icterus. During the systemic examination, the patient was not oriented to time or place but was oriented to the person. Meningeal signs were negative, and there was no focal neurological deficit. On respiratory examination, there was wheezing in the bilateral lower zones of the lung. The rest of the examination findings were within normal limits.

Investigations

As presented in Table 1, his complete blood count showed anemia with a hemoglobin of 8.6 (13.5, 14.5 mg/dL) and a total leukocyte count of 4.380 x 103/uL (4-11 x 103/uL). The peripheral smear showed normocytic normochromic red blood cells (RBCs) with mild anisocytosis, a normal white blood count (WBC) with mild left shift, and normal platelets. Serum ferritin was high at 1,650 ng/mL, and the rest of the anemia workup (reticulocyte count, stool for occult blood, and Coomb's test) was normal. The kidney function test was within the normal range. Liver function was suggestive of a cholestatic pattern of liver injury attributed to antitubercular-related drug injury, which the patient took for one month. Ultrasonography of the whole abdomen was normal. HIV positivity was reconfirmed (a repeat test was done in our institution for documentation of evidence as there were no lab reports of HIV positivity attached in the discharge summary of the prior admitted hospital).

**Table 1:** Baseline investigations at admission. g/dL: Grams per deciliter; uL: Microliter; mg/dL: Milligram per deciliter; ng/ml: Nanogram per milliliter; IU/L: International units per liter; mEq/L: Milliequivalent per liters; SGOT: Serum glutamic-oxaloacetic transaminase; SGPT: Serum glutamic-pyruvic transaminase; ALP: Alkaline phosphatase; GGT: Gamma-glutamyl transferase; TLC/DLC: Total cell count/differential cell count; CBNAAT: Cartridge-based nucleic acid amplification test; VDRL: Venereal Disease Research Laboratory; KOH: Potassium hydroxide; PCR: Polymerase chain reaction; BAL: Bronchoalveolar lavage; MIC: Minimum inhibitory concentration

Lab Parameters	Patient’s Report
Hemoglobin (g/dL) (normal: 12-16 g/dL)	8.6
Total leukocyte count (×10^3^/uL) (normal: 4-11×10^3^/uL)	4.38
Platelets (109/L) (normal: 150-400×10^9^)	152
Bilirubin(mg/dL) (normal: 0.1-1.2 mg/dL)	3.8
SGOT/SGPT(IU/L) (normal: 10-40 IU/L/29-33 IU/L)	66/120
ALP (normal: 40-120 U/L)	247
GGT (normal: 5-40 U/L)	56
Serum protein (g/dL) (normal: 6-8 g/dL)	5.2
Albumin (g/dL) (normal: 3.5-5.5 g/dL)	3
Serum urea (mg/dL) (normal: 20-40 mg/dL)	42
Creatinine (mg/dL) (normal: 0.5-1.1 mg/dL)	0.7
Sodium (mEq/L) (normal: 136-145 mEq/L)	138
Potassium (mEq/L) (normal: 3.5-5.2 mEq/L)	4.2
Serum ferritin normal: 40-300 ng/mL)	1650
CSF sugar (normal: 50-80 mg/dL)	62
CSF protein (normal: 15-45 mg/dL)	37
CSF TLC/DLC (normal: < 5/hpf)	Acellular
CSF CBNAAT	Negative
CSF India ink	Negative
CSF KOH stain	Negative
CSF VDRL test	Negative
CSF PCR for toxoplasma	Negative
CSF BioFire	Negative for Cryptococcus, Mycobacterium, Syphilis, and Toxoplasma
Serum cryptococcal antigen	Negative
BAL culture	Acinetobacter baumannii -10^5 ^CFU/mL intermediate sensitive to colistin (MIC < 0.5)
BAL AFB	Negative
BAL CBNAAT	Negative (repeat came positive)
BAL gomori methamine stain	Negative
USG abdomen	Normal
CEMRI brain	Normal

**Figure 1:**
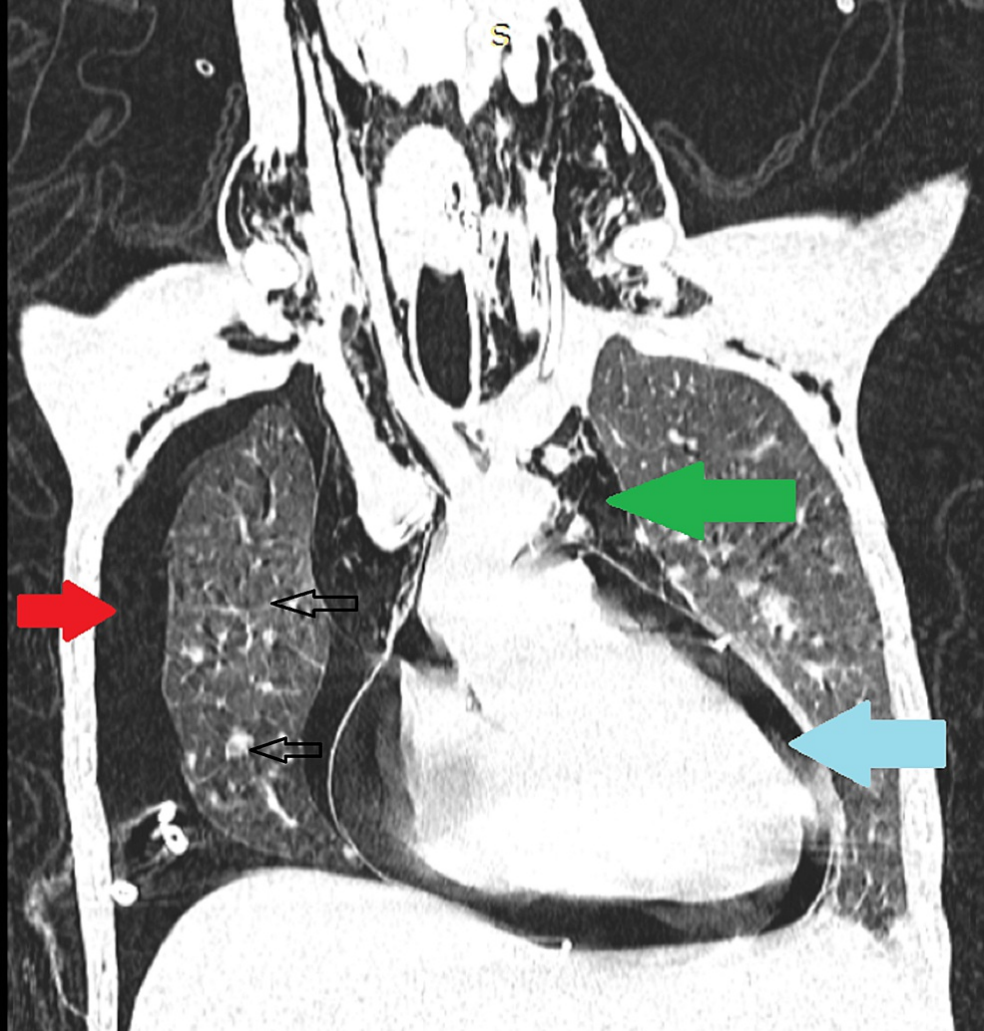
Contrast-enhanced computed tomography (CECT) chest showing the lung window of the patient. There is a presence of pneumothorax (red arrow), pneumomediastinum (green arrow), and pneumopericardium (blue arrow). Gross lung changes can be seen in the form of nodules and ground glassing in the background.

CD4 count was 13 cells/mm^3^. CSF was normal and was negative for Cryptococcus, Mycobacterium, Syphilis, and Toxoplasmosis. Contrast-enhanced MRI brain was also normal. Bronchoalveolar lavage was done, but it was negative for Mycobacterium tuberculosis (acid-fast bacilli (AFB) stain and cartridge-based nucleic acid amplification test (CBNAAT)), Mycobacterium avium complex (acid-fast staining), and pneumocystis pneumonia (Gomori methamine silver stain). Other non-tuberculous mycobacteria and acid-fast bacilli cultures were not tested due to economic issues of the patient party), as well as Pneumocystis jirovecii. Cultures of BAL were positive for Acinetobacter baumannii (105 CFU/mL), which was intermediate sensitive to colistin. Fungal culture and galactomannan were negative. Contrast-enhanced computed tomography (CECT) thorax and abdomen were done, which showed active infective changes in bilateral lungs (images could not be retrieved). Multiple ground-glass attenuations and solid centrilobular nodules were seen in the bilateral lower lobes. Some of them showed trees in bud patterns. There was a lobulated, air-filled cyst of size 15 x 13 mm in the superior segment of the left upper lobe. After three days, there was increased oxygen requirement, hypotension, and tachycardia, and repeat imaging (Figure 1) showed right pneumothorax, pneumomediastinum, and pneumopericardium. The BAL sample was also repeated, which came positive for Mycobacterium with CBNAAT positivity and was sensitive to rifampicin.

Treatment

The patient was admitted for the management of the drug-induced liver injury. He was started on modified ATT with the SLE (streptomycin of 750 mg IM), levofloxacin of 750 mg IV), and ethambutol of 800 mg per oral) regimen. Colistin (nebulization of 150 mg twice daily) was initiated when initial BAL cultures came positive for Acinetobacter.

Prophylaxis for pneumocystis with cotrimoxazole (160/800 mg once daily) and atypical mycobacterium with azithromycin (1.2 g once weekly) was given. Highly active antiretroviral therapy (HAART) was initiated with tenofovir, lamivudine, and dolutegravir (TLD) regimen simultaneously with modified ATT treatment. CECT thorax showed ground-glass opacities and air-filled cysts, following which cotrimoxazole (160/800 mg thrice daily) was started in therapeutic doses as CT thorax had features suggestive of pneumocystis pneumonia. However, the patient developed increasing oxygen requirements, hypotension, and tachycardia due to pneumothorax, pneumopericardium, and pneumomediastinum. Immediate intercostal drainage insertion and pericardiocentesis were done. Empirical treatment was initiated for cytomegalovirus (CMV) with ganciclovir (250 mg twice daily intravenously). Mechanical ventilation was provided due to respiratory failure.

Repeat BAL was done as the clinical status of the patient was deteriorating, and the sample came positive for Mycobacterium tuberculosis (CBNAAT positive, sensitive to rifampicin).

Outcome

The patient developed worsening septic and obstructive shock and severe metabolic acidosis despite all measures and went into cardiac arrest from which he could not be revived.

## Discussion

Spontaneous pneumothorax is a frequent complication in HIV-infected patients. The major causes identified were bacterial pneumonia, Pneumocystis jiroveci pneumonia (PJP), and pulmonary tuberculosis [4]. PJP was the more common cause in patients with a CD4+ lymphocyte count <200 cells/mL or with acquired immune deficiency syndrome (AIDS) [4]. The incidence of spontaneous pneumothorax in HIV patients was higher in the pre-HAART era [5,6]. The most common radiographic abnormalities in pneumocystis pneumonia are diffuse, bilateral interstitial, or alveolar infiltrates. Thin-walled cysts, or pneumatoceles, are seen in 10-20% of cases. In our patient, with a CD4 count of 13, there were indications suggestive of pneumocystis pneumonia, such as ground glass opacities and air-filled cysts. However, we were unable to obtain microbiological evidence confirming pneumocystis pneumonia. The occurrence of spontaneous pneumomediastinum (SPM) has been linked to a diverse range of lung conditions, including bronchial asthma, dermatomyositis, systemic sclerosis, and other connective tissue disorders. Additionally, associations with diabetic ketoacidosis, interstitial lung disease, influenza-like syndrome, cancer-related complications, chronic obstructive pulmonary disease (COPD, excluding asthma), broncholithiasis, tuberculosis, anorexia nervosa, cystic lung disease, achalasia cardia, PCP/HIV, myocardial infarction, and acute respiratory distress in newborns due to 21-hydroxylase deficiency have been noted [7]. Macklin in 1944 showed that SPM is thought to be caused by an alveolar rupture, which can happen because of high intra-alveolar pressure, low perivascular pressure, or both [8]. The frequency of concomitant pneumothorax reported is 6-11% [7]. In a comprehensive review conducted by Cheng et al., they observed 11 cases of pneumomediastinum in individuals infected with HIV. Out of these cases, nine were associated with pneumocystis pneumonia, while two cases exhibited co-infections. Among the co-infections, one involved Staphylococcus aureus, and the other presented a combination of the Nocardia asteroides complex, Legionella pneumophila, and Streptococcus. In these cases, no influenza or PCP co-infection was reported [8].

Pneumopericardium is another rare entity due to blunt or penetrating trauma or pericardial infections. The mechanism of air entry into the pericardial cavity, known as the “Macklin effect,” is postulated by Macklin. This phenomenon results from an alveolar rupture, leading to a sudden increase in intrathoracic pressure. The elevated pressure causes air to leak into the pericardium through different pathways. If there is a pleuropericardial tear, the air may enter the pericardium via the pleural cavity, especially when the visceral pleura is disrupted, causing pneumothorax. Alternatively, the air can be tracked through the lung interstitium, following the perivascular planes of pulmonary vessels. This may result in the migration of air into the mediastinum, neck, retroperitoneum, and eventually the pericardium. The Macklin effect describes the intricate process by which air can travel from ruptured alveoli to the pericardial cavity through these anatomical pathways [9].

In our case report, the initial CECT thorax had demonstrated an air-filled cyst, which could have ruptured, resulting in the above conditions.

## Conclusions

To summarize, the co-occurrence of spontaneous pneumothorax, pneumomediastinum, and pneumopericardium in an HIV-infected tuberculosis patient is an extremely rare and complicated clinical presentation. This case emphasizes the importance of a multidisciplinary approach to managing such cases, emphasizing the importance of early diagnosis, aggressive treatment of underlying infections, and close monitoring to avoid life-threatening complications. More research is needed to better understand the pathophysiological mechanisms underlying this rare trio of immunocompromised conditions, with the goal of ultimately improving patient care and outcomes.
